# Conditions for Nutritional Care of Elderly Individuals with Dementia and Their Caregivers: An Exploratory Study

**DOI:** 10.3390/nu17061007

**Published:** 2025-03-13

**Authors:** Paola Sarmiento-González, Maria Elisa Moreno-Fergusson, Luz Indira Sotelo-Diaz, Gabriela Rabe Caez-Ramírez, Laura Nathaly Ramírez-Flórez, Beatriz Sánchez-Herrera

**Affiliations:** 1 Nursing and Rehabilitation School, Campus Puente del Común, Universidad de La Sabana, Chía 250001, Colombia; paola.sarmiento1@unisabana.edu.co (P.S.-G.); mariae.moreno@unisabana.edu.co (M.E.M.-F.); laurarafl@unisabana.edu.co (L.N.R.-F.); 2 EICEA Department of Gastronomy, Campus Puente del Común, Universidad de La Sabana, Chía 250001, Colombia; indira.sotelo@unisabana.edu.co; 3 Engineering School, Campus Puente del Común, Universidad de La Sabana, Chía 250001, Colombia; gabriela.caez@unisabana.edu.co

**Keywords:** nurses improving care for health system elders, nutritional status, feeding behavior, geriatric nursing, dementia

## Abstract

**Background/Objective**: Although the context, personal conditions, and caregivers’ abilities influence the nutrition of older people with dementia, adequate parameters are not always applied to identify these conditions. The aim of this study was to characterize the nutritional care needs of older people with dementia and their caregivers. **Method**: This descriptive exploratory study was conducted in Colombia. An intentional sample included 73 elderly individuals with dementia and 73 caregivers. This study described the participants’ characteristics with the GCPC-UN-D survey. Their nutritional conditions include medical history, objective tests, and interviews. We used the Edinburgh Scale to evaluate elderly feeding behavior and the QUALID tool to evaluate their quality of life. This study measured caregiver competence using the CUIDAR tool. **Results**: Adults of 78.8 years on average, with low to middle socio-economic status, low education levels, and multiple comorbidities, have adverse well-being and support conditions, except for the spiritual component. These adults have visible nutritional issues including low muscle mass indices (47.9%), muscle mass levels (arm 61.6%; calf 58.9%), and vitamin D levels (50.7%), with high cholesterol levels (57.9%) and altered hematocrit and red blood cell counts (46.4%). These adults required supervision (41%) or help (23%) for their nutrition. Caregivers were predominantly women with an average age of 32.4 years, with moderate caregiving competences (70.43%), experienced high caregiver burden (83.6%), and had low orientation in nutritional management (30.1%). **Conclusions**: Elderly individuals with dementia had significant nutritional and feeding problems. Their caregivers lacked adequate conditions to ensure quality care. These dyads need a strategy to improve their healthcare experience.

## 1. Introduction

The world is experiencing a rapid and progressive aging trend [1]. By 2050, the highest concentration of elderly individuals will be in countries with low and middle development levels [1]. This global aging trend is associated with an increased incidence of chronic diseases and cognitive decline. Among the elderly, there is up to a 10% incidence of diseases that cause dementia, and its prevalence doubles for every five additional years of life [2].

Nutrition and feeding are related to dementia in several ways. On the one hand, a healthy diet is a preventive measure against dementia [3], and on the other, individuals with dementia are at a higher risk of malnutrition and difficulties during meals [4,5,6,7,8]. Their nutritional status also affects the progression of their decline [9,10]. Furthermore, nutritional problems are linked to the perceived burden of caregivers of individuals with dementia [11]. When caregivers lack the necessary conditions to ensure proper nutrition, they perceive a higher burden and often opt for lower-quality diets [12].

Interventions aimed at the nutrition and feeding of elderly individuals with dementia and their caregivers have proven useful, highlighting the need to understand the specific characteristics of those with dementia and their caregivers [13,14]. However, there are no known studies on the nutritional conditions of elderly individuals with dementia and their caregivers residing in the Andean region, where cases of cognitive decline and dementia are reported at twice the rate found in other regions [15]. While there are important research advances on the nutritional care of older adults with severe cognitive deficits driven by the roles of their caregivers, these are limited in Colombia. Care for this population group has particular socio-cultural and economic conditions, and health systems differ significantly from those where there is evidence. It is crucial to close this knowledge gap as inadequate nutrition in this population leads to adverse health and functionality outcomes and increases the burden of care for their caregivers. This study aims to characterize the conditions for nutritional care of a group of elderly individuals with dementia and their primary caregivers in the Andean region of Colombia as a basis for planning future care interventions that address their needs.

## 2. Materials and Methods

### 2.1. Study Design and Participants

This is a quantitative, descriptive exploratory cross-sectional study. We selected this design because the characterization of needs requires an initial systematic approach to a phenomenon, in this case, the nutritional care needs of older people with dementia and their caregivers. This study describes the particularities of this specific phenomenon. It is part of a larger research project titled Nurturing Neurons, 2022–2025.

The population consisted of institutionalized elderly individuals with dementia and their primary caregivers residing in Colombia. We used intentional sampling for convenience. Given the exploratory nature of this study, we used a sample size of 146 individuals, including 73 elderly individuals with dementia and their 73 caregivers from the central Andean region of Colombia, which encompasses both urban metropolitan areas and highly rural municipalities. Our sample and type of sampling help us secure a population with which communication can be maintained, and is easily accessible in case our findings lead to the development of future projects for their benefit.

### 2.2. Data Collection

In older adults with dementia, this study addressed conditions for care, nutritional guidelines, objective nutritional status parameters, and subjective parameters identified through interviews. In formal caregivers, it also addressed caregiving conditions and caregiving competences. All data were collected by nurses with experience in geriatrics and gerontology.

The conditions for care were described using the GCPC-UN-D survey [16]. This survey outlines care characteristics in three dimensions through 21 items addressing both the person with dementia and the caregiver: (1) sociodemographic situation, (2) perceived burden and support, and (3) use of information and communication technologies (ICTs) for care. The nurses surveyed individuals with dementia or their legal representatives and their primary caregivers. We assessed ICT adoption based on parameters proposed by Melengüe [17].

We created a protocol based on the clinical experience of the researchers and parameters reported in the literature to evaluate the nutritional condition of elderly individuals with dementia [18,19,20]. Eleven experts from various disciplines (nutrition, medicine, endocrinology, and nursing) with over 3 years of experience in geriatrics or gerontology validated the protocol. The final version included nutritional guidelines, objective parameters, and interviews.

We derived nutritional guidelines from instructions given by physicians or nutritionists in medical records.

Objective parameters included the following: (1) anthropometric measures of height, waist, hip, arm, and right calf circumference, as well as heel–knee height, measured in centimeters, and weight measured in kilograms using a previously calibrated electronic scale; (2) grip strength assessed with a Takei 5401 hand dynamometer on the dominant arm; (3) hydration level and oral condition assessed through physical examination; (4) blood analyses and biochemical tests: complete blood count, total cholesterol, glycated hemoglobin, albumin, folic acid, vitamins B12 and D. We conducted the analysis of these parameters using standardized criteria for this population [21,22,23].

This study included two tools to guide the interviews: (1) The first was the Edinburgh Feeding Evaluation in Dementia Scale (EdFED) [24,25]. Through caregiver interviews, the EdFED detects the difficulties elderly individuals with dementia experience during meals. EdFED has 10 questions rated from 0 to 2 based on whether they are absent, infrequent, or frequent. These include the need for supervision, physical help, food spillage, refusal or gestures interfering with eating, such as turning the head, refusing to open the mouth, spitting, or difficulty swallowing. (2) The second was the Dementia Quality of Life Scale (QUALID) [26,27]. QUALID consists of 4 items rated from 1 to 5 based on the frequency of occurrence in the past week, evaluated with the support of the primary caregiver, including expressions of joy, sadness, crying, and discomfort.

We used the CUIDAR tool (short version) to assess caregiver competence [28]. This tool consists of 20 questions rated from 0 to 3 on a Likert scale and measures overall caregiver competence in the following dimensions: knowledge, uniqueness, well-being conditions, anticipation, and support network. Total scores between 82 and 100 indicate high competence, from 47 to 81 moderate competence, and 46 or less low competence.

### 2.3. Statistic Analysis and Methodological Rigor

This study included a detailed description and correlation between the main variables in order to explore their characteristics in more detail. We collected individual results for every participant in this study and analyzed them by statistical descriptors, Pearson correlation, and heat correlation maps among gender, age, institution, and demographic attributes with blood chemistry, well-being, and autonomy indicators [29]. We performed an analysis using R Studio v.2024.09.0 with the packages ggplot2 for visualization and corrplot, which provides dedicated functions for plotting correlation matrices as heatmaps [30].

We controlled extraneous variables in the study design through a careful review of existing literature and expert knowledge for protocol validation, the inclusion of individuals with similar characteristics, and the selection of statistical techniques for their exploration.

### 2.4. Ethical and Environmental Considerations

This study received institutional ethical approval (No. ENF-602021). It adhered to ethical principles for research involving human subjects as expressed in the Declaration of Helsinki [31] and national regulations [32]. It included consent from the legal representatives of elderly individuals with dementia and their caregivers. This study met environmental protection criteria [33] by minimizing the use of paper, disposable products, and energy consumption.

## 3. Results

The study group of elderly individuals ranged from 60 to 95 years old, with an average age of 78.8 years. Their caregivers ranged from 19 to 65 years old, with an average age of 32.4 years. Their care characteristics are described in detail in Table 1 (see Table 1).

Medical diagnoses reported in medical records indicate high cognitive impairment in 28.8% of cases. Among these, 52.3% have Alzheimer’s disease, 19.0% have impairment associated with mental health disorders, 9.5% are associated with intellectual disabilities, 4.7% have Lewy body dementia, 4.7% have frontotemporal dementia, 4.7% have vascular dementia, and 4.7% have dementia associated with Pick’s disease.

Nutritional guidance was documented in 30.1% of the records, as detailed in Table 2 (see Table 2).

The objective nutritional status parameters are reported in the following tables (see Table 3 and Table 4).

The following tables illustrate the results of the interviews regarding elderly individuals with dementia (see Table 5 and Table 6).

Finally, the caregivers’ competency was reflected in average percentages across categories and overall, except for knowledge, where we found a low level: knowledge, 46.7%; uniqueness, 70.5%; instrumentation, 73.3%; enjoyment of well-being conditions, 81.3%; anticipation, 74.3%; support network, 72.0%; and overall, 70.43%.

**Figure 1:**
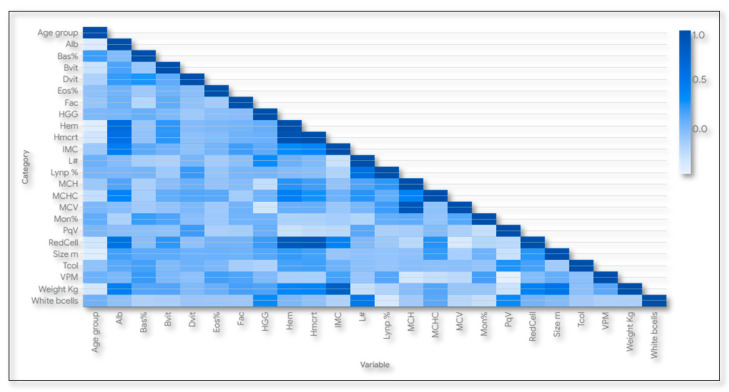
Correlation matrix heatmap for blood indicators. Source: Study data, 2025.

The correlation analysis for older adults results among age, sex, body mass index (IMC), weight, and size with blood chemistry indicators (Figure 1) showed a nonlinear correlation for age group with size (−0.39), hemoglobin (−0.39), red cells (−0.32) and weight (−0.37). The negative Pearson correlation indicated a decrease in those variables with age increase. Weight indicated a nonlinear correlation with size (0.55), hematocrit (0.38), red cells (0.41), and albumin (0.42). BMI presented a nonlinear correlation with albumin (0.44) and hemoglobin (0.32). The positive Pearson correlation indicated an increase in those variables when increasing weight and BMI. Glycosylated hemoglobin had a nonlinear correlation with white blood cells (0.31). Finally, hemoglobin was nonlinearly correlated with mean corpuscular hemoglobin (0.43) concentration and a linear correlation with hematocrit (0.95).

**Figure 2:**
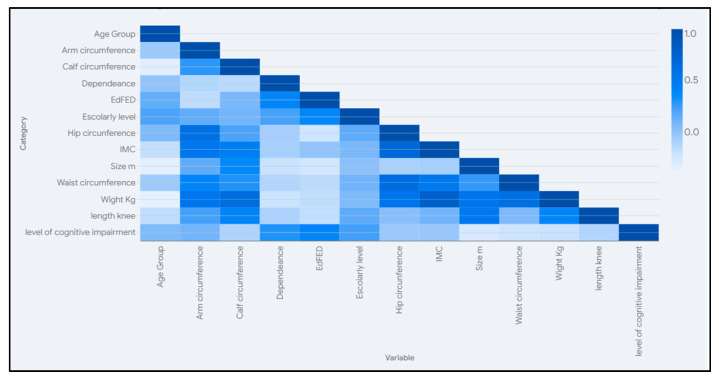
Correlation matrix heatmap for anthropometric and well-being variables. Source: Study data, 2025.

The correlation between the age of older people with dementia and sex and objective tests showed more dispersion in weight and waist perimeter in older groups as shown in Figure 2. There were significant nonlinear moderated correlations by sex with the level of cognitive impairment (−0.38), indicating a tendency for females to have higher levels of cognitive impairment compared to males. Also, the sex variable showed a moderate positive relationship with waist circumference (0.36) and a linear trend with size (0.6), indicating a tendency for males to have larger waist circumferences and size compared to female older adults.

Weight showed a linear correlation with waist circumference (0.61), hip circumference (0.55), calf circumference (0.64), and arm circumference (0.55). Hip circumference indicated a linear correlation with IMC (0.70), arm circumference (0.61), and waist circumference (0.63). On the other hand, a nonlinear correlation was found between EdFed and the scholarly level (0.38), level of cognitive impairment (0.40), and dependence level (0.40).

## 4. Discussion

This study characterized the nutritional care needs of older adults with dementia and their caregivers. Its findings confirm the need for a comprehensive assessment to attend to the nutritional care issues of elderly individuals with dementia and to support their caregivers [18].

The age of these elderly individuals exceeds the global average life expectancy by six years. This corroborates that there is a higher risk of developing dementia as age increases, especially when there are comorbidities [34]. In this sample of older adults, most comorbidities associated with cognitive impairment, including cardiovascular and endocrine diseases, can relate to the presence of cognitive deterioration [35,36]. Evidence points to a higher prevalence of both malnutrition and cognitive impairment among elderly individuals with cardiovascular problems, such as the “cold and dry” phenotype of heart failure, characterized by low perfusion and low congestion at rest [37,38,39]. This phenomenon could be explained by the reduction in cardiac output leading to chronic cerebral hypoperfusion. This change activates the neurohumoral system, systemic inflammation, decreased microvascular circulation, cerebral hypoxia, and alteration of the blood–brain barrier. Overall, the risk of stroke and cognitive decline is increased. In turn, impaired cognitive function, as noted above, increases the risk of malnutrition, creating a vicious cycle.

The age of individuals with dementia is 2.4 times greater than that of their caregivers. Other studies report on age differences between those with dementia and their caregivers [40]. However, this study found a larger gap, which might be explained by the fact that all caregivers are formal caregivers.

The gender distribution shows a predominance of females both among individuals with dementia and their caregivers, reinforcing the notion that dementia affects women more, both directly and indirectly [2].

The educational level could not be assessed for almost a quarter of these older people, mainly because some of them cannot vouch for their background and there are no records of their educational history. Of the other 75%, almost half only have a high school education or less, while almost all caregivers have technical or higher education. These results are consistent with studies conducted in the same region and other regions [41,42]. Low educational level may explain the previous occupations reported by these elderly individuals and is associated with their vulnerable socioeconomic condition. This also supports previous findings that link low socioeconomic status and low levels of early education with a higher risk of dementia [43,44,45].

An earlier study reports a positive association between caregiver education and their understanding of the condition and the care required for individuals with dementia [46]. However, in this case, the caregivers’ competencies did not reflect this finding, despite their studies and level of experience in the field. Several authors have associated the low level of caregiver competencies with a care burden perception [47,48,49,50,51,52]. These caregivers need new educational and support strategies to care for older people with dementia who have frequent feeding problems.

Our findings ratify that Alzheimer’s disease is the most frequent diagnosis in people with severe cognitive impairment worldwide [2]. However, the high number of nonspecific diagnoses we found once more shows a challenge in caring for specific groups of elderly patients with dementia [53]. Moreover, we found a lack of adequate guidance for their nutritional care despite its importance given the malnutrition and dietary complications associated with these conditions [6,54,55,56].

The quality of life for these institutionalized elderly individuals appears to indicate that most are calm, smile frequently, experience low levels of sadness, and have low levels of crying and discomfort. Their low levels of physical, psycho-emotional, and social well-being, and the low perception of support in these areas, may be related to their isolated lives. Although most receive financial support, the lack of a stable partner, widowhood, and the distance from family could impact their condition [57]. These living conditions and perceived support call for a review and improvement of their current situation [58]. Conversely, the relatively high level of perceived spiritual well-being and their high commitment to religious practice reported by most elderly individuals are supportive factors that should be explored further [59].

Caregivers, on the other hand, report high levels of physical and psycho-emotional well-being and moderate levels of social and spiritual well-being. Although most lack a stable partner, they feel a high level of family support but report low levels of psychological, religious, economic, and social support, which may contribute to increased caregiver burden and high stress levels. The high risk of caregivers of individuals with dementia experiencing low well-being and quality of life has been well documented; however, most of these studies raise nonformal caregiver issues [60,61]. Therefore, it is important to consider social support strategies to reduce the perceived burden [62].

Caregivers do not perceive a high level of commitment to their faith, although most identify as Catholic or Christian. This finding reaffirms the reported association between religious commitment and caregiver competence [59].

The adoption of ICT for care shows that elderly individuals have greater familiarity with television and radio, while caregivers report familiarity with the phone, television, and the internet (84.8%). This finding opens possibilities for specific support for these dyads. However, it will be necessary to develop orientation strategies to ensure the quality of information selection and delivery [63,64,65]

Regarding the objective assessment of elderly individuals, the body mass index (BMI) reflects nutritional problems due to deficiency in one-quarter of the elderly group, and excess in 2.7%. It is important to recognize that despite the usefulness of these measures, the effectiveness of this index has been debated as a universal indicator of nutritional status in elderly individuals [66,67]. In this case, arm circumference measurements reflect the same proportion of individuals with nutritional problems, while calf circumference slightly increases in the group with malnutrition due to deficiency. Conversely, 93.75% of the cases have an elevated waist-to-hip ratio. These findings reinforce the reported association between low muscle mass, high fat proportion, and cognitive decline in elderly populations [68]. One-quarter of the elderly group have a low BMI, although all of them have normal waist circumferences and normal waist–hip ratios. These apparently paradoxical results may be explained because older adults naturally lose muscle mass, which is denser than fat and may have an impact on overall weight reduction, even if abdominal fat remains normal [69]. Therefore, in this case, it is crucial to consider the individual’s overall health and medical history when interpreting these measurements.

Dehydration is frequent in these population groups [70]. However, hydration was normal for most elderly subjects (83.1%), which could be related to adequate fluid intake and climate stability typical of the region. A larger group (45.1%) showed problems with their oral condition, confirming the association found between oral health and cognitive problems [71,72]. However, it is important to conduct a study comparing the oral health of this population when they do not have cognitive impairments.

Handgrip strength is recommended by several authors as a relevant measure of nutritional status in elderly individuals and is related to cognitive decline [73,74]. However, in this case, there was difficulty in measurement due to the inability of these elderly individuals to follow the instructions.

Clinical laboratory tests showed that hemoglobin A1c was normal for 73.9% of the elderly individuals, while 13.0% were prediabetic and 8.6% diabetic, one-third of whom were poorly controlled. Elevated levels of hemoglobin A1c have been reported in individuals with dementia of any cause [75].

Vitamin B12 was normal for most of the group (85.5%), as was folic acid (94.2%), which could question whether adequate vitamin B and folate are protective factors against dementia [76]. Conversely, there was a marked deficiency of vitamin D (50.7%) in this group, ratifying that this vitamin, which regulates neurotransmitters and has neuroprotective properties, tends to be low in individuals with cognitive impairment. This is particularly relevant in relation to Alzheimer’s disease, although the benefits of supplementation for its prevention and treatment need further study [77].

Although serum albumin is responsible for most of the plasma’s antioxidant properties and is associated with reduced neurodegeneration and cognitive decline in elderly individuals [78], and serum deficiency is linked to appetite problems in this population, 78.2% of the group had normal albumin levels, with even elevated levels in 13.0% of cases. Cholesterol was elevated in 37.6%, which contradicts McFarlane et al., who found that this is not an indicator of cognitive decline risk [79], but adheres to a recent large cohort study with more than 1.8 million people and two decades of follow-up that ratified earlier evidence and suggests including LDL cholesterol to the list of modifiable risk factors for dementia [80].

Anemia has been associated with an increased risk of dementia from all causes, with causal relationships between hemoglobin and red blood cell distribution width, and the risk of cognitive decline [81]. In this group, hemoglobin was normal in 66.6% of the elderly and deficient in 24.6%, which supports these findings. Similarly, hematocrit percentage, mean corpuscular volume, hematocrit, and mean platelet volume showed U-shaped associations with dementia risk [81], which are not supported by these findings.

In contrast, there are no observable differences regarding total leukocyte populations, platelet counts, the neutrophil-to-lymphocyte ratio, the platelet-to-lymphocyte ratio, or PCR levels between elderly individuals with dementia and those with intact cognition, leading to the conclusion that these do not seem to be specific biomarkers for this type of diagnosis [82]. The same finding applies to the platelet volume observed [83].

A limitation of this study is the scope to which its results can be applied given its sample and sampling selection. It could be inferred that community-dwelling, non-institutionalized individuals are subject to the conditions and capabilities of their caregivers, making their prognosis more uncertain. However, these results may be useful for guiding the development of an intervention aimed at a similar population.

## 5. Conclusions

This exploratory study establishes that this elderly group of people with dementia is vulnerable and needs specialized nutritional care. Their physical deterioration is evidenced by low muscle mass, vitamin D deficiency, and hematological abnormalities. They also have a high dependence on assistance with feeding, and they require supervision and help. On the other hand, formal caregivers experience a high care burden, and they lack the tools and guidance to address nutritional care adequately. Both patients and caregivers need an integrated intervention that promotes nutritional care quality, improves caregiving skills, and addresses the well-being of older people with dementia.

## Figures and Tables

**Table 1:** Characteristics of the elderly with dementia and caregivers.

Dimension	Assessed Aspect			Subject	
				Person with Dementia	Caregiver
				%	%
Sociodemographic profile	Sex	Male		37.0	9.6
		Female		63.0	90.4
	Education level	No education		2.7	0
		Primary		38.4	0
		Secondary (High school)		9.6	1.3
		Technical or Technologist		11.0	97.2
		Professional		12.3	1.3
		Postgraduate		2.7	0
		Non-Applicable or No Response (N.A./N.R.)		23.3	0
	Housing	Origin	Andean Region	93.2	83.5
			Other region	4.1	13.6
			Other country	0	2.7
			N.A./N.R.	2.7	0
		Place of residence	Andean Region	100	100
		Type of residence	Particular	2.7	100
			Institution	97.3	0
		Residential Area	Urban	86.3	4
			Rural	13.7	67.1
	Marital Status	Single		34.2	54.7
		Married		23.3	6.8
		Separated		4.1	0
		Common-law		4.1	38.3
		Widow(er)		30.1	0
		N.A./N.R.		4.1	0
	Occupation (previous, in the case of older adults)	Housekeeper		8.21	0
		Employee		8.21	100
		Independent		11.0	0
		Student		0	0
		Agriculture		19.2	0
		Driver		5.5	0
		Others		6.8	0
		N.A./N.R.		41.1	0
	Health Status	Cognitive Sphere	Intact	0	100
			Altered	100	0
		Level of Functionality	Functional	0	100
			Moderate dependence	72.6	0
			High dependence	27.4	0
		Medical Diagnoses	Healthy	0	81
			With medical diagnosis	100	19
			Severe cognitive impairment (CI)	100	0
			CI + cardiovascular disease	60.3	0
			CI + endocrine disease	28.8	0
			CI + gastrointestinal disease	5.5	0
			CI + respiratory disease	4.1	0
			CI + musculoskeletal disease	12.3	0
			CI + sensory organ disease	4.1	0
			CI + neurological disease	11.0	0
			CI + psychiatric disease	13.7	0
			CI + renal disease	1.4	0
			CI + dermatological disease	2.7	0
	Socioeconomic status	Low (1–2)		60.3	67.1
		Medium (3–4)		26.0	33
		High (5–6)		12.3	0
		N.A./N.R.		1.4	0
	Religion	Type	Catholic	86.3	85
			Christian	5.5	8.2
			Agnostic	0	1.4
			Atheist	4.1	5.5
			Other	0	0
			N.A./N.R.	4.1	0
		Level of commitment	High	61.6	0
			Medium	23.3	60.3
			Low	11.0	24.7
			N.A./N.R.	4.1	0
Perception of burden and support	Provides care for people with dementia	Since diagnosis		N.A.	23.3
		After diagnosis		N.A.	76.7
	Sole caregiver	Yes		N.A.	0
		No		N.A.	100
	Daily hours required for care	11 h or less		4.1	19.2
		12 to 23 h		19.2	54.7
		24 h		75.3	24.7
		N.A./N.R.		1.4	0
	Type of caregiver	Family member		N.A.	0
		Formal caregiver			100
	Time spent as a caregiver	Less than 1 year		N.A.	20.5
		1 to 2 years			12.3
		2 to 4 years			9.6
		More than 4 to 10 years			34.2
		More than 10 years			23.3
	Previous caregiving experience	No experience		N.A.	15
		With experience			85
	Perception of burden or support	High		17.8	35.6
		Medium		16.4	48
		Low		52.1	16.4
		N.A./N.R.		13.7	0
		Psychological support	0	17.8	45.2
			1	9.6	1.4
			2	13.7	13.7
			3	38.4	24.7
			4	12.3	15
			N.A./N.R.	8.2	0
		Family support	0	4.1	11
			1	1.4	1.4
			2	24.7	2.7
			3	24.7	19.1
			4	24.7	65.7
			N.A./N.R.	8.2	0
		Religious support	0	23.3	64.3
			1	2.7	11
			2	5.5	6.8
			3	23.3	2.7
			4	38.3	15
			N.A./N.R.	6.8	0
		Economic support	0	6.8	49.3
			1	0	4.1
			2	0	8.2
			3	57.5	22
			4	27.3	16.4
			N.A./N.R.	8.2	0
		Social support	0	13.6	19.1
			1	8.2	6.5
			2	19.1	15
			3	35.6	35.6
			4	15	23.2
			N.A./N.R.	8.2	0
	Perception of well-being	Physical well-being	0	1.4	0
			1	6.8	2.7
			2	23.3	12.3
			3	41	48
			4	16.4	37
			N.A./N.R.	11	0
		Psycho-emotional well-being	0	1.4	0
			1	11	0
			2	20.5	13.6
			3	46.5	48
			4	11	34.3
			N.A./N.R.	9.5	0
		Social well-being	0	1.4	1.4
			1	15	6.8
			2	22	15
			3	34.2	35.6
			4	19.1	41
			N.A./N.R.	8.2	0
		Spiritual well-being	0	4.1	1.4
			1	5.5	8.2
			2	4.1	19.1
			3	30	26
			4	48	45.2
			N.A./N.R.	8.2	0
Level of Technology Use for Care		TV		61.4%	88.4%
		Radio		28.4%	64.5%
		Computer		12.0%	74.6%
		Phone		17.7%	96.7%
		Internet		12.8%	84.8%

Source: Study data, 2025.

**Table 2:** Nutritional guidance for elderly individuals with dementia.

Type of Nutritional Guidance		%
No orientation		69.9
Enriched diet	High in iron and fiber with liquefied protein	1.40
Hypoglycemic diets	Hypoglycemic	12.5
	Hypoglycemic, dairy-free, no pork	
	Hypoglycemic, seafood allergy	
	Hypoglycemic, soft proteins and thick juices	
	Hypoglycemic, liquefied proteins	
	Liquefied proteins	
	Hypoglycemic, high in fiber, no grains	
	Hypoglycemic, cardiovascular alteration diet	
	Hypoglycemic, red meat up to 2 times/week	
Low-sodium diets	Low sodium	2.8
	Low sodium, no dairy, and fluid restriction	
Diet for cardiovascular issues	Cardiovascular alteration diet	5.5
	Cardiovascular alteration diet, no soups	
Other food restrictions	Unsweetened juices	5.2
	No acidic foods, no dairy	
	No juices, liquefied protein	
	No dairy, no grains	

Source: Study data, 2025.

**Table 3:** Anthropometric measurements, oral condition, and hydration of elderly individuals with dementia.

Indicator	Results in %		
	Deficit/Low	Normal	Excess/High
BMI	24.7	52.1	15.1
Arm circumference	61.6	34.2	2.7
Calf circumference	Moderate 30.1	41.1	0.0
	Severe 28.8		
Hydration	13.7	84.9	0.0
Oral condition	0.0	15.1	N.A.
	11.0	30.1	N.A.
	32.9	N.A.	N.A.
Waist circumference	0.0	38.4	58.9
Waist-to-hip ratio	0.0	5.5	Moderate 24.7
			Severe 57.5
Grip strength	21.9	4.1	0.0

Source: Study data, 2025.

**Table 4:** Clinical laboratory results for elderly individuals with dementia.

Type of Test		Results in %		
		Low	Normal	High
Glycated hemoglobin		0	73.9	Prediabetic 13
				Diabetic 8.6 (controlled 2.8)
Vitamins	Vitamin B12	1.01	85.5	0
	Vitamin D	50.7	44.9	0
	Folic acid	0	94.2	14
Albumin		2.8	78.2	13.0
Total cholesterol		0	57.9	Limit 21.7
				High 15.9
Complete blood count	Hemoglobin	24.6	66.6	4.3
	Hematocrit	39.1	53.6	2.8
	Red blood cell count	39.1	53.6	2.8
	Mean corpuscular volume	2.8	85.5	7.2
	Mean corpuscular hemoglobin	2.8	44.9	47.8
	Mean corpuscular hemoglobin concentration	0	50.7	44.9
	Red cell distribution width	0	71.0	24.6
	White blood cells	7.2	88.4	0
	Neutrophils %	1.4	89.5	4.3
	Neutrophils (absolute count)	0	94.2	1.4
	Lymphocytes %	1.4	91.3	2.8
	Lymphocytes (absolute count)	4.3	91.3	0
	Monocytes %	10.1	76.8	8.6
	Monocytes (absolute count)	27.5	68.1	0
	Eosinophils %	0	89.5	5.7
	Eosinophils (absolute count)	44.9	50.7	0
	Basophils %	0	94.2	1.4
	Basophils (absolute count)	0	94.2	0
	Platelet count, automated method	4.3	94.2	11.5
	Mean platelet volume	8.6	75.3	11.5

Source: Study data, 2025.

**Table 5:** Feeding problems of elderly individuals with dementia.

Problem Assessed	Performance Level %		
	Normal	Somewhat Committed	Very Committed
1. Need for close supervision to eat	59	8	33
2. Need for physical assistance to eat	77	7	16
3. Food spillage during eating	70	12	18
4. Food remaining on the plate after finishing a meal	67	19	14
5. Refusal to eat	85	11	4
6. Turning the head while eating	85	10	5
7. Refusal to open their mouth	92	5	3
8. Spitting out food	95	0	5
9. Not closing the mouth, allowing food to fall out	92	3	5
10. Difficulty swallowing food	84	11	5

Source: Study data, 2025.

**Table 6:** Quality of life of elderly individuals with dementia.

Condition Assessed	Occurrence Level %				
	1	2	3	4	5
Smiling	49.2	16.9	11.3	12.7	9.8
Sadness	45.1	22.5	12.7	12.7	7.0
Crying	63.4	15.5	11.3	8.4	1.4
Discomfort	39.4	36.6	14.1	1.4	8.4

Source: Study data, 2025.

## Data Availability

The data presented in this study are available on request from the corresponding author. Data are not publicly available due to the authors being interested in contacting different organizations that are interested in the same topics.
